# Evaluation of Combining Several Statistical Methods with a Flexible Cutoff for Identifying Differentially Expressed Genes in Pairwise Comparison of EST Sets

**DOI:** 10.4137/bbi.s431

**Published:** 2008-05-01

**Authors:** Angelica Lindlöf, Marcus Bräutigam, Aakash Chawade, Olof Olsson, Björn Olsson

**Affiliations:** 1 School of Humanities and Informatics, University of Skövde, Box 408, 541 28 Skövde, Sweden; 2 Department of Cell and Molecular Biology, Göteborg University, Box 462, 403 20 Göteborg, Sweden

**Keywords:** expressed sequence tags, statistical methods, differentially expressed genes

## Abstract

The detection of differentially expressed genes from EST data is of importance for the discovery of potential biological or pharmaceutical targets, especially when studying biological processes in less characterized organisms and where large-scale microarrays are not an option. We present a comparison of five different statistical methods for identifying up-regulated genes through pairwise comparison of EST sets, where one of the sets is generated from a treatment and the other one serves as a control. In addition, we specifically address situations where the sets are relatively small (~2,000–10,000 ESTs) and may differ in size. The methods were tested on both simulated and experimentally derived data, and compared to a collection of cold stress induced genes identified by microarrays. We found that combining the method proposed by Audic and Claverie with Fisher’s exact test and a method based on calculating the difference in relative frequency was the best combination for maximizing the detection of up-regulated genes. We also introduced the use of a flexible cutoff, which takes the size of the EST sets into consideration. This could be considered as an alternative to a static cutoff. Finally, the detected genes showed a low overlap with those identified by microarrays, which indicates, as in previous studies, low overall concordance between the two platforms.

## Background

Expressed sequence tag (EST) sets originate from randomly picked clones in a cDNA library and generate information about transcript abundance (Lindlof, 2003; Nagaraj et al. 2007). This information can be used for analyzing gene expression patterns from different conditions and has been valuable in the discovery of biologically interesting genes. Gene expression levels in unbiased cDNA libraries can be estimated by using the cognate frequencies of gene transcripts. The variation in frequency of ESTs sampled from different libraries can be used for detecting genes appearing to be differentially expressed in a biological experiment (Audic and Claverie, 1997; Claverie, 1999; Greller and Tobin, 1999; Man et al. 2000; Romualdi et al. 2003).

During the last years, several statistical methods have been proposed for detecting differentially expressed genes in multiple EST sets (Audic and Claverie, 1997; Claverie, 1999; Greller and Tobin, 1999; Romualdi et al. 2001; Man et al. 2000; Ruijter et al. 2002; Stekel et al. 2000). In such experiments there are at least two sets and the aim is to investigate whether a gene is significantly differently expressed in one set in comparison to the other(s). This approach has frequently been used for identifying tissue-specific genes, and is also of importance when addressing the differences between normal and pathological conditions. It is also a method used when comparing wild-type specimens with stressed ones or the differences in gene expression between different crop varieties (Bräutigam et al. 2005; Fei et al. 2004; Gulick et al. 2005; Schmitt et al. 1999; Strausberg et al. 2001).

The efficiency of several statistical methods used for this purpose has previously been evaluated (Romualdi et al. 2001). We intend to present another comparison of these statistical methods. However, in this study, the focus is different from that in previous studies. Here, we aim to address the differences between a control and treatment condition, i.e. a pairwise comparison between a normal and stressed condition, where the compared sets are relatively small (~2,000–10,000 ESTs) and the total number of ESTs in the two sets might differ. In addition, our aim is to investigate if the detection of up-regulated genes requires a rigid statistical test or if a more simple measurement would be sufficient, and if the combined outcome from several methods will improve the overall results.

The comparison of the statistical methods is first performed on simulated data sets, since this provides a controlled environment where the methods can be properly tested. The observations from these tests are thereafter applied to the experimental data from cold stressed *Arabidopsis thaliana* plants.

Therefore we are presenting the results from the comparison of five different methods for identifying up-regulated genes in two EST sets, the χ^2^ test (χ^2^) (Ugoni and Walker, 1995), Fisher’s exact one-sided and two-sided test (Fone and Ftwo, respectively) (Blevins and McDonald, 1985), the test developed by Audic and Claverie (1997) (AC), and a method consisting of simply calculating the difference in relative frequency between the two sets (Diff) (Fei et al. 2004; Mochida et al. 2006; Ogihara et al. 2003; Pavy et al. 2005; Lynn et al. 2003).

The results from the simulation studies show that the methods are comparable, but produce slightly different results, which indicate differences in their sensitivity. In addition, the simulation studies show that the combination of AC, Fisher’s and Diff increases the number of detected up-regulated genes, and that the size of the sets is important when setting a proper cutoff on the test values. Therefore, we introduce a flexible cutoff, which takes this issue into consideration. In the simulation studies, this resulted in lower variability in the percentage of true positives (genes detected that are truly up-regulated in the treatment), when we compared the results from different simulations with a large variation in sample size. Using the flexible cutoff a similar level of true positives was reported from all comparisons, despite the difference in size between treatment and control sets.

We tested the combination of the AC, Fisher’s and Diff methods together with the flexible cutoff on experimental data derived from cold-stressed *Arabidopsis thaliana* plants, using non-stressed plants as a control. The results of this study show that the percentage of true positives was relatively low (~25%). This can be a result of various factors; for instance, there are many steps included when progressing from the biological experiments to the detection of up-regulated genes and each step is concerned with difficulties that will affect the results. Additionally, we used a collection of cold induced genes identified from microarray studies as a reference (Hannah et al. 2005). Previous studies have shown a low overlap between the two techniques (van Ruissen et al. 2005; Haverty et al. 2004; Kavsan et al. 2007) and the results indicate that the same applies for this study.

## Results and Analysis

### Statistical methods

**Figure 1:**
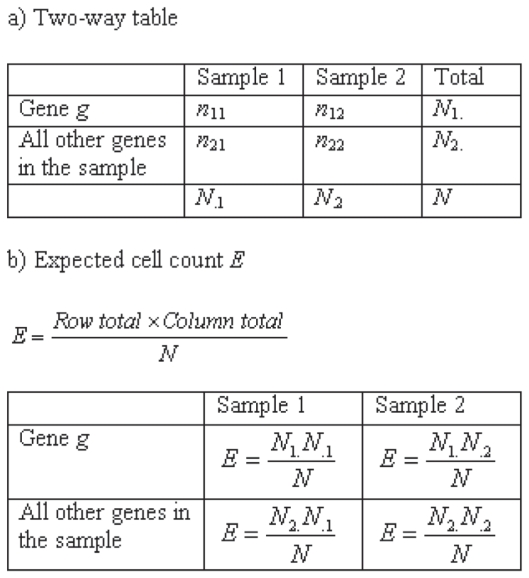
In **a**) it is shown that EST frequencies can be arranged in a two-way table, where Sample 1 and 2 refer to EST set 1 and 2, respectively. *n*_1,j_ is the number of ESTs originating from gene *g* in set 1 and 2, respectively, *n*_2,j_ is the summed number of ESTs for all other genes in set 1 and 2, respectively, *N*_i_. is the sum of row *i*, *N*._j_ the sum of column *j* and *N* is the grand total. **b**) The expected cell count *E* can be computed from the two-way table, by multiplying the row total and column total for a cell in the two-way table and thereafter dividing by the grand total.

The expression levels derived from an EST experiment are represented by integers, which correspond to the number of ESTs that have been matched to a specific gene. In order to identify differentially expressed genes, this expression measurement is compared between two or more sets, commonly originating from different conditions, such as wild-type vs. treated plants. The key question is whether a gene is differentially expressed in one condition in comparison to the other. Since the expression values can be arranged in a two-way table (Fig. 1a), standard statistical tests, such as the χ^2^ test and Fisher’s exact test (Claverie, 1999), have been applied and used for detecting significant differences between the experimental conditions.

However, there has been some criticism against the application of these two tests to this type of data. It has been suggested that Fisher’s exact test is too conservative and therefore excludes a large proportion of biologically interesting genes (Audic and Claverie, 1997; Romualdi et al. 2001). The χ^2^ actually tests whether the conditions differ as a whole, rather than for each gene individually, as desired (Audic and Claverie, 1997; Claverie, 1999). Instead, Audic and Claverie, (1997) developed a pairwise test that was shown to be more sensitive and less conservative than Fisher’s exact and χ^2^ tests as it takes the specific characteristics of EST data into consideration. In addition, they showed that EST frequency distributions could be approximated by the Poisson distribution and incorporated this into the test, along with the size of each set, since the compared EST sets may differ in size.

In the comparisons conducted here we choose to focus on genes that are up-regulated in the treatment, since in experiments where transcripts have been sampled from a stress-situation the majority of the ESTs will originate from up-regulated genes. For example, when the plant is subjected to cold stress, there is a redistribution of which biological processes are active and inactive. In this situation, energy producing processes, for example, are shut down, such as photosynthesis and development pathways. Instead, processes needed for protecting the cell against freezing are activated, such as the production of cryoprotectants, sugar and prolines (Beck et al. 2007; Chinnusamy et al. 2007). Genes involved in these signaling pathways will become overrepresented in EST sets derived from cold stress cDNA libraries and, consequently, be considered as up-regulated in the treatment when compared to a control.

Here, we applied the general χ^2^ 2 × 2 test (χ^2^), the Audic and Claverie (1997) one-sided test (AC), and Fisher’s exact one- and two-sided test (Fone and Ftwo, respectively). We also included the one-sided version of Fisher’s exact test, in contrast to previous studies where the two-sided version has been used. This was done since the interest here was in identifying up-regulated genes in one set in comparison to the other and we wanted to investigate whether the one-sided test might be more appropriate than the two-sided test. Additionally, we also tested the approach by simply calculating the difference in relative frequency (Diff), since relative frequencies are commonly used when clustering EST expression profiles from different libraries (Fei et al. 2004; Pavy et al. 2005; Henry et al. 2004; Lynn et al. 2003; Marvanova et al. 2002; Ogihara et al. 2003).

Since we were interested in genes that are up-regulated in the treatment, we wanted to exclude genes detected as differentially expressed but overrepresented in the control condition. In order to do so, we only regarded those genes that had a higher relative frequency in the treatment EST set than in the set generated from the control condition.

### Simulation data

**Figure 2:**
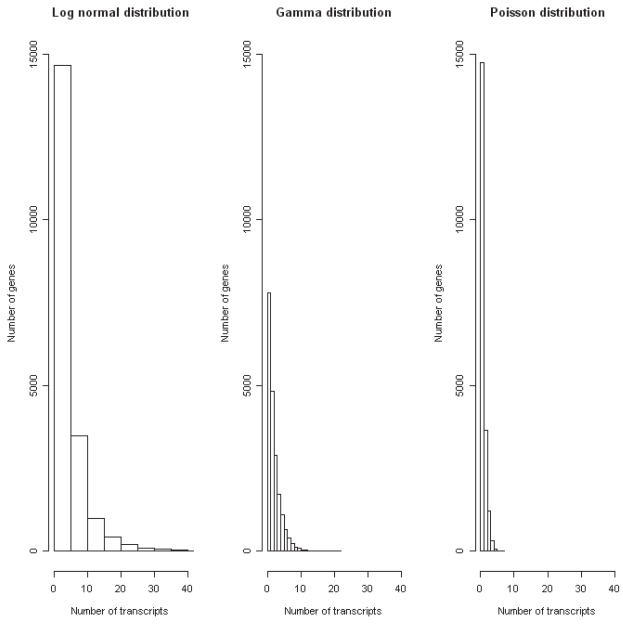
Histograms of the Poisson, Log normal and Gamma distributed expression levels for 20,000 genes, respectively. The distributions were derived using the R statistical language (see Methods for more details) and sampling 20,000 individuals (i.e. genes). For the Log normal distribution a mean *μ* = 1 and standard deviation *σ* = 1 was used, for the Gamma distribution a shape *α* = 1 and scale *β* = 2.0 was used, and for the Poisson distribution a shape *λ* = 1 was used.

In real experiments, gene expression levels based on an EST set are represented by integers. Furthermore, highly expressed genes are more likely to be sampled from the cDNA library than weakly expressed ones, resulting in a larger number of transcripts for those genes. However, most genes are weakly or moderately expressed, and therefore most genes are represented by a small number of transcripts. This commonly results in most genes being sampled only once or twice, some being sampled more than twice, and very few being sampled a large number of times. This distribution can be modeled, for example, by the Log normal or the Gamma probability distribution (Fig. 2). In addition, the Poisson distribution has previously been suggested to be the most adequate for describing EST sampling data (Audic and Claverie, 1997; Claverie, 1999).

Based on these observations, we created pseudo cDNA libraries following any of the three distributions, from which we draw sets with varying sample sizes. In more detail, we created pseudo cDNA libraries containing around 1,000,000 clones relating to 20,000 genes, and where the transcript abundance of a gene (denoted by *x*) followed either:

- a Log normal distribution with mean *μ* = 1 and standard deviation *σ* = 1
  (1) $$f(x;\mu ,\sigma )=\frac{1}{x\sigma \sqrt{2\pi }}e^{-{\left(\text{ln}x-\mu \right)}^{2}/2\sigma^{2}}\;\text{for }x>0,$$
- a Gamma distribution with shape *α* = 1 and scale *β* = 2.0
  (2) $$f(x;\alpha ,\beta )=x^{\alpha -1}\frac{e^{-x/\beta }}{\beta^{\alpha }\Gamma (\alpha )}\text{for }x>0,$$
- a Poisson distribution with shape *λ* = 1
  (3) $$f(x;\lambda )=\frac{e^{-\lambda }\lambda^{x}}{x!}\;\;\;\text{for x}>0$$

From each of these three pseudo libraries we simulated the transcript abundance of 4,000 genes (20% of the total 20,000) as up-regulated with a specified fold change (See Methods for more details on the library construction).

We generated several pseudo libraries for the treatment condition, to test different sample sizes as well as different levels of fold-change. Genes simulated as up-regulated were randomly chosen in each pseudo library, and, in addition, the fold change for the same gene varied in the different libraries.

It has previously been established that genes with a high expression level in combination with a high fold change are easily detected by the statistical methods (Romualdi et al. 2001). However, when the expression level decreases as well as the fold change, it gets more difficult to separate true differentially expressed genes from false positives. By varying the genes simulated as up-regulated, choosing both weakly, moderately and highly expressed genes, as well as their fold-change, this issue is taken into consideration.

### Simulation studies—simple model

In the first round of simulations the fold-change followed a normal distribution *N*(*m*, *sd*) with *m* = 2 or *m* = 10, and *sd* = 1, i.e. each gene was randomly simulated with an up-regulation of 2 or 10 fold changes, with a slight spread set by the standard deviation. This was done merely to investigate the effect of having a relatively low fold-change in comparison to a high one. In these simulations, 2,000 and 10,000 ESTs were sampled from each of the pseudo libraries.

**Figure 3:**
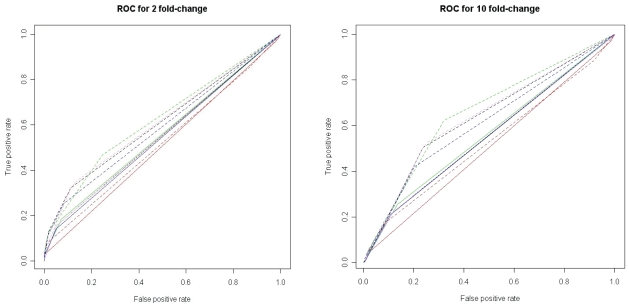
ROC curves for the identification of up-regulated genes. Genes were simulated as having either a 2-fold change (left) or a 10-fold change (right). Solid and dashed lines indicate 2,000 and 10,000 sampled ESTs, respectively, and the colors refer to the different statistical methods. For these figures the transcript abundance followed a log normal distribution. For the other distributions, see supplemental figure S1. Curves show the difference in relative frequency. Black: Diff, red: χ^2^, green: AC, violet: Fone, blue: Ftwo.

Commonly a cutoff is set on the test value for the statistical methods that are used and genes are detected according to the cutoff. We tested different values of the cutoff (Table 1) and derived ROC-curves (Receiver Operating Characteristic) based on these cutoffs (Fig. 3 and S1). The ROC curve is a plot of the sensitivity versus 1-specificity when using a binary classifier in combination with increasing or decreasing the threshold of the discriminator. Here, the classifier is whether a gene is up-regulated or not and the discriminator is one of the different statistical methods. The plot can be used for investigating the performance of a test—the closer the ROC curve is to the upper left corner the higher is the overall accuracy of the test.

Since we recorded which genes were simulated as up-regulated, the sensitivity (the number of up-regulated genes present in the treatment set that are correctly detected by the method), the specificity (the number of non-differentially expressed genes present in the treatment set that are correctly disregarded by the method), and the number of true positives (the number of true up-regulated genes among those detected by the method) could be calculated.

The results confirm the conclusions made by Romualdi et al. (2001) that the efficiency of the methods increases when the differential expression increases (Fig. 3), since the ROC curves indicate improved performance with larger fold-change values. However, the size of the EST set is also of importance, since the separation of true positives from false ones is improved when a high fold-change is used in combination with a large sample set, compared to the situation when a high fold-change is combined with a smaller sample set. Further, we can see that the different transcript distributions generate almost identical results (Fig. 3, S1 and S2).

Interestingly it was noted that the Diff method performs as well as the other statistical methods. The χ^2^ test performs slightly worse than the other methods. Fisher’s test has previously been criticized and not considered appropriate for this type of data (Audic and Claverie, 1997; Romualdi et al. 2001). On the other hand, this test should be used when the sample size is too small for the χ^2^ test. Here, we can see that both Fisher’s one- and two-sided tests perform as well as or better than the other methods. Additionally, the one-sided test yielded a slightly better result than the two-sided test.

### Simulation studies—more complex model

**Figure 4:**
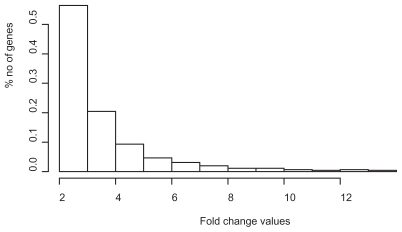
The histogram shows the distribution of fold change values (FC), where FC ≥ 2, taken from a real microarray experiment (see text and Methods for more details).

In the second round of simulations the fold-change values followed an exponential probability distribution with *λ* = 1, to get a more realistic model. The choice of probability distribution was based on a histogram of gene expression values from a real microarray experiment, where it could be observed that the values approximately followed this distribution (Fig. 4). The microarray experiment was conducted on cold-stressed *Arabidopsis thaliana* plants, where the plants had been stressed by cold temperature at 4 °C and compared to non-stressed plants. The microarray experiment was generated by AtGenExpress (see Acknowledgement) and downloaded from TAIR (Garcia-Hernandez et al. 2002). The histogram shows all genes with at least a 2 fold-change at time point 0.5 h.

**Figure 5:**
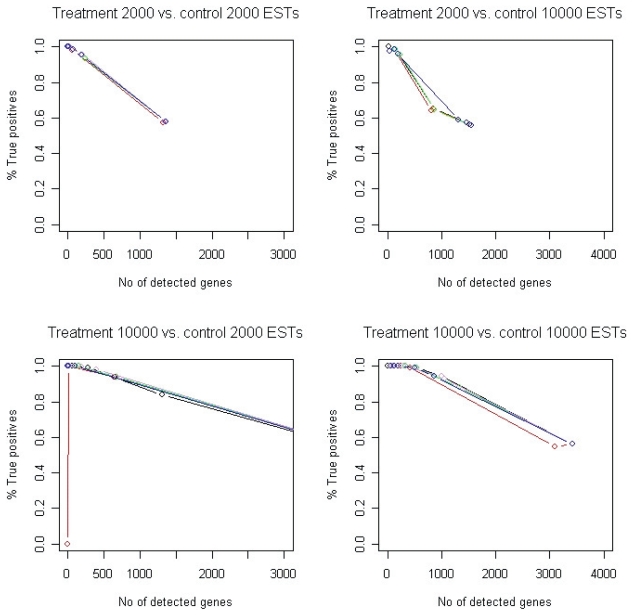
These plots show the percentage of true positives detected by the different statistical methods versus the total number of detected genes according to different cutoffs. Here, the transcript abundance followed a log normal distribution and the size of the EST sets differed in the treatment and control set. Black: Diff, green: AC, violet: Fone, blue: Ftwo, red: χ^2^. The circles correspond to different cutoffs according to Table 2 for each statistical method.

As in the above simulation study, both 2,000 and 10,000 clones were sampled from each generated pseudo library. After assessing the rate of true positives, it was revealed once again that all methods perform on a comparable level (Fig. 5, S2 and S3). The χ^2^ test performs slightly worse than the other methods when the size of the EST sets increases (see Fig. 5 for the χ^2^ test when 10,000 ESTs from treatment are compared with 10,000 ESTs from control). Additionally, we can see that for very low *p*-values no genes are detected as up-regulated (as an example, see Fig. 5 and the χ^2^ test when 10,000 ESTs from treatment are compared with 2,000 ESTs from control).

In previous studies it has been reported that the different methods are not entirely overlapping in the results, which indicates differences in their sensitivity (Fei et al. 2004). This can also be deduced from Figure 5, since the methods report varying numbers of detected genes and different percentages of true positives at comparable cutoffs. For example, when studying the comparison for which 10,000 ESTs were picked from both the treatment and control library the AC test reports 544 detected genes with 99% true positives when using a cutoff of 0.1. This is comparable to Fone when using a cutoff of 0.1. However, this test reports 298 detected genes with 100% true positives.

### Data mining on simulated data

Since the results from the methods differ slightly, with varying numbers of detected genes at different cutoff levels, it can be difficult to decide which cutoff to use and which method to rely on. It may also be the case that the best results are obtained when using a combination of methods. The problem, of course, is to decide how the different methods should be combined in order to obtain the best results. To investigate this issue we applied data mining techniques on the results, and derived decision trees which might guide the choice of the optimal combination of statistical methods.

For this simulation study we 1) sampled a random number of ESTs from the treatment and control pseudo library, 2) thereafter applied the different methods to the generated EST sets, and 3) computed test values from all statistical methods for each pairwise comparison. This sampling and pairwise comparison procedure was conducted 20 times; for each sampling the genes simulated as up-regulated were randomly chosen. In addition, the fold-change set for each up-regulated gene was also randomly chosen from the underlying exponential probability distribution.

The concatenated results from the 20 comparisons together with the recorded class, i.e. whether a gene was simulated as up-regulated or not, were used as input to the decision tree induction algorithm J48 in the Weka software (Frank et al. 2004). Trees were thereafter generated using the entire training set (no cross-validation was used; see Figure S4 for an excerpt of input data to J48). Furthermore, this whole procedure was repeated five times for each transcript distribution, i.e. five trees were generated for each distribution, where each tree was based on data generated from 20 different simulations.

**Figure 6:**
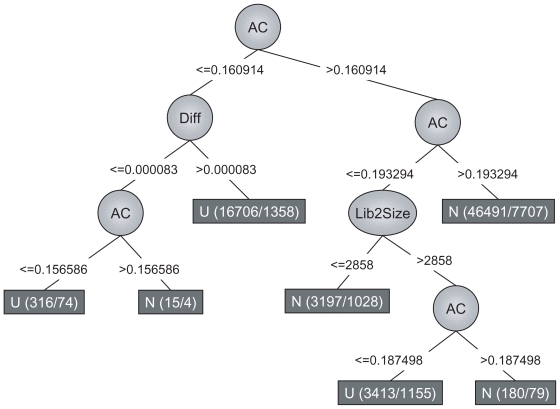
Example of a decision tree generated by the induction algorithm J48, available in the Weka program (Frank et al. 2004). Each leaf rectangle represent a class—either up-regulated (U) or non-differentially (N) expressed. The circles and the expressions on the edges indicate different attributes and conditions that must be fulfilled, respectively. The nodes labeled ‘AC’ and ‘Diff’ represent the statistical methods AC and Diff, respectively and the values on the associated edges correspond to *p*-values (for AC) and difference in relative frequency (for Diff). The node ‘Lib2Size’ refers to the size of the control set (i.e. library 2 in the comparison). The values inside the squared boxes indicate the total number of genes that have been detected and the number of genes with that class, respectively.

The decision trees generated by J48 varied quite extensively among the different training sets (Fig. 6 and S5), which implies that the algorithm has difficulties in making a correct classification and is strongly dependent on the training set used. This conclusion is also supported by a relatively moderate percentage of correctly classified instances (on average ~69%). The complexity of the trees varied from being very simple, including only a few nodes, to more complex with 10–20 nodes. The trees generated when using a Poisson distribution were in general less complex than the others (data not shown).

These observations indicate a lot of noise in the data and, hence, it is difficult to identify up-regulated genes from the EST data. This most likely relates to the large number of non-affected genes in the sets. Many of the genes in the pseudo libraries were not simulated as up-regulated, but will nevertheless be picked up in the sampling of the library due to a high or moderate expression value. This also relates to the real situation, i.e. the majority of the genes expressed in a cell do not participate in the stress response, but are likely to be picked from the cDNA library if they have a high or moderate expression level. Consequently, these genes will become false positives. The number of false positives highly depends on the number of up-regulated genes and the level of fold-change for those genes. If the up-regulated genes have a much higher fold-change, then the number of false positives will decrease.

However, there are some common characteristics among the trees, such as the AC test almost always being the root node. This test also seems to have a critical value around 0.18–0.19 in the simulation studies, where values larger than this threshold need to be supported by an additional method. In addition, the size of the EST set sometimes appears as a node in the decision tree, thus implying that the sample size is of importance when setting the cutoff. For example, in Figure 6 the attribute ‘Lib2Size’ is represented by a node in the tree, which indicates that the size of the control library (i.e. library 2 in the comparison) is used for separating up-regulated from non-differentially expressed genes.

### Testing and applying rules

Based on an analysis of the trees, a range of simple rules were tested on a new round of simulated data. Since size appeared as a node in the decision trees, we additionally tested different combinations of sample sizes. The sample size was divided into the categories small (2,000–4,000 ESTs) medium (5,000–7,000 ESTs) and large (8,000–10,000 ESTs), and a random number of transcripts were sampled within each range.

We tested all combinations of sample sizes from each pseudo library, i.e. first a small set from the treatment vs. a small set from the control library, second, a small set from the treatment vs. a medium-sized set from the control, and so on. For each combination the sampling was repeated three times, each time randomly choosing genes as up-regulated, as well as setting their fold-change value to a randomly chosen one from the underlying exponential probability distribution.

Initially, different cutoffs on the test values were implemented and tested (Table 2, rule R1-R22). For example, for the AC test four different cutoffs (*p*-value={0.16, 0.18, 0.19, 0.29}) were used to detect up-regulated genes. For each cutoff used, the percentage of true positives as well as the sensitivity was calculated for all rounds of simulations (i.e. three repeats of all combinations of different sample sizes). The results from these simulations can be viewed in Figure 8 where the left boxplots show the percentage of true positives and the right ones the sensitivity for each method.

It can be deduced from the boxplots in Figure 8 that a more relaxed cutoff results in a lower percentage of true positives. On the other hand, a larger number of up-regulated genes are detected (higher sensitivity). For example, an AC cutoff of 0.16 results in an average of ~70% true positives and ~30% of the total number of up-regulated genes (sensitivity). When increasing the cutoff to 0.20, this yields ~65% true positives and ~40% sensitivity (compare the boxplots for 0.16 and 0.20 for the AC method in Fig. 8). Considering true positives, the best results are obtained with Fone or Ftwo using a cutoff of *p* ≤ 0.1, which results in an average of ~99% true positives. However, the sensitivity is very low, with only ~2% on average. It can also be noticed that the results from the AC test in general vary less among the different sets, with fewer outliers and more compact boxes, regardless of the cutoff used. This indicates that the sizes of the ESTs sets are less important in the comparisons, than for the other methods. For the others, the percentage of true positives and the sensitivity begins to vary greatly when the cutoff becomes more relaxed (with the exception for Diff at *D* ≥ 0.0001 and χ^2^ at *p* ≤ 0.5). The results from these methods depend on both the number of sequenced ESTs and the number of up-regulated genes present in the sets.

**Figure 7:**
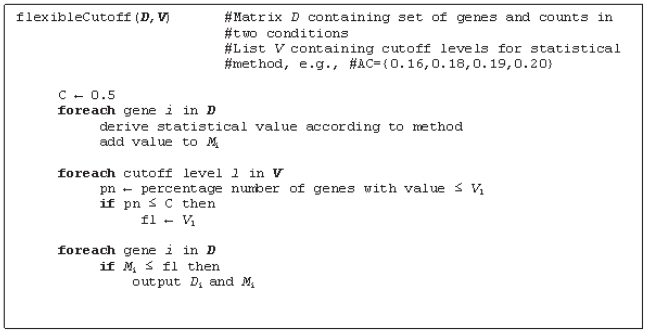
Pseudocode for the algorithm of using a flexible cutoff for detecting up-regulated genes in a treatment vs. a control set.

**Figure 8:**
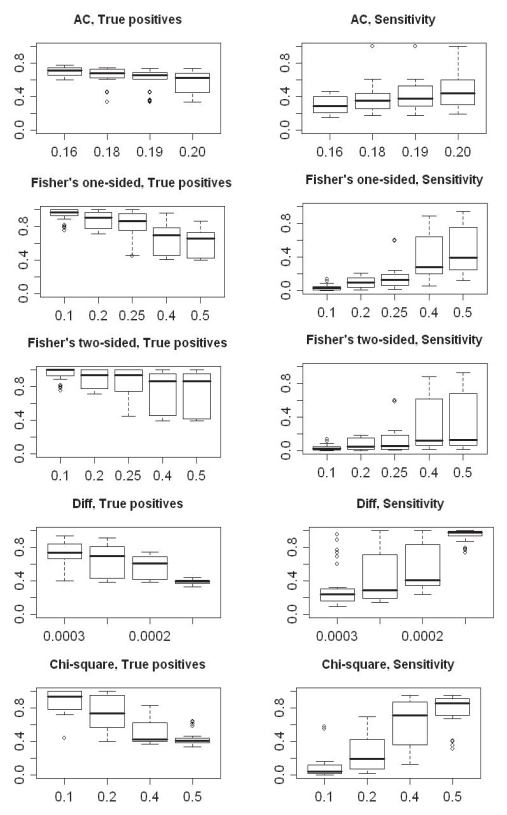
Boxplots illustrating the percentage of true positives detected (plots on the left) and sensitivity (plots on the right) for each statistical method, when using different cutoffs for deriving up-regulated genes (see Table 2 for an outline of cutoffs used). On the x-axis are the different cutoffs and on the y-axis the percentage of true positives and sensitivity, respectively.

Since the results from the tests of using a specified cutoff and the decision trees implied that the cutoff was dependent on the sample size, we incorporated this characteristic in yet another round of rules and simulations (Table 2, rule R23–R27). The difficulty here is that the sample size varies among the EST sets, where commonly one set is large and the other one much smaller. We therefore introduce the use of a flexible cutoff *C*, which is a percentage that is used for determine the cutoff on the test values (i.e. *p*-values or the difference in relative frequency), and is based on the number of detected genes from a comparison and their corresponding test values. For different possible cutoffs on the test values, e.g. *p* = {0.001, 0.005, 0.01, 0.05, 0.1}, the percentage number of genes with ≤*p* is calculated. For example, in one comparison 12% of the detected genes have a *p* ≤ 0.001, 34% a *p* ≤ 0.005 and 52% a *p* ≤ 0.01. The level of *C* determines which test value cutoff will be used in deriving up-regulated genes, and this will be the test value that have been derived for <*C*% of the genes (pseudo code shown in Fig. 7). Referring to the example, if C = 50%, the test value cutoff will be 0.005, since the percentage number of genes with *p* ≤ 0.005 is 34%, which is less than *C*.

We tested four different levels on the flexible cutoff: *C* = 0.1 (10%), 0.3 (30%), 0.5 (50%) and 0.7 (70%), and derived the percentage of true positives and sensitivity for each method using the same simulated data as in the above study.

**Figure 9:**
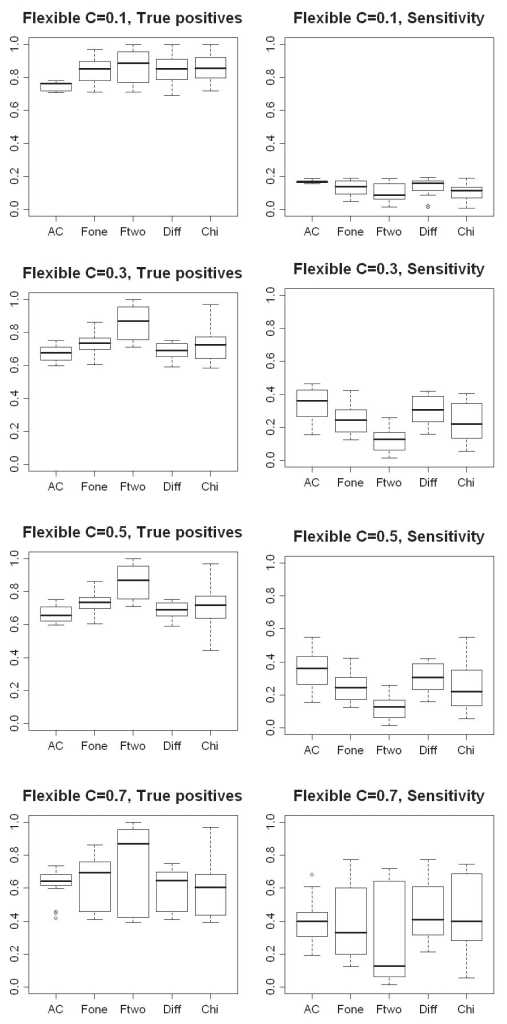
Boxplots illustrating the percentage of true positives detected (left plots) and sensitivity (right plots) when applying the flexible cutoff for detecting up-regulated genes in the two EST sets. On the x-axis are the different cutoffs and on the y-axis the percentage of true positives and sensitivity, respectively.

For the AC method the flexible cutoff only affects the results slightly when changing the value on *C* (see Fig. 9, true positives and sensitivity for AC). The average is ~70% and ~40% for percentage of true positives and sensitivity, respectively. The only exception is when using a highly stringent value, *C* = 0.1, for which the percentage of true positives reaches ~80% and the sensitivity ~20%.

For the remaining methods, the largest difference appears when the cutoff is increased from 0.1 to 0.3 or from 0.5 to 0.7. When a cutoff of either 0.3 or 0.5 is used there are almost similar results in both percentage of true positives and sensitivity. The only exception is for the χ^2^ test, for which the number of false positives greatly increases when the cutoff is increased to 0.5. This method also shows the highest variability in the results, for some generated EST sets the percentage of true positives ranges from very high to very low when considering the same cutoff. One example is the χ^2^ test when *C* is set to 0.3, for which the percentage of true positives range from 99% to 59%.

**Figure 10:**
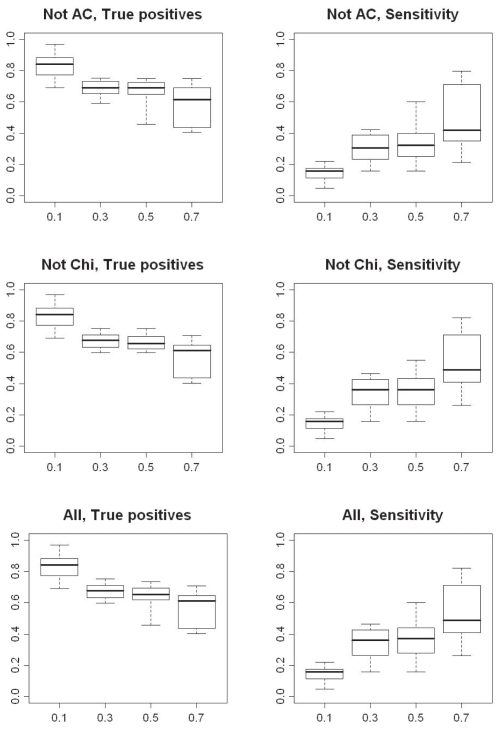
Boxplots illustrating the percentage true positives detected and sensitivity (lower plot) when combining 1) χ^2^, Fone and Diff methods with a flexible cutoff 2) AC, Fone and Diff methods with a flexible cutoff and 3) all methods with a flexible cutoff for detecting up-regulated genes in two EST sets. On the x-axis are the different levels of the flexible cutoff used and on the y-axis the percentage true positives and sensitivity, respectively.

However, the different methods do not produce exactly the same results, even when a flexible cutoff is used. Therefore, we tested an additional number of rules where the different statistical methods were combined (Table 2, R18–R24). In this case we tried the combination of ‘leaving one out’, i.e. all methods except one were combined with the flexible cutoff. This was done to investigate whether one method had a larger impact on the results than any of the others (Fig. 10). Here, almost identical results were generated for all combinations, with the only exception being when the χ^2^ test was left out. Therefore, only the results for leaving out the AC and χ^2^ tests are shown in Figure 10. When χ^2^ is left out identical results are produced for cutoff 0.3 and 0.5 regarding true positives, which ranges from 77% to 59%. The range for the sensitivity is slightly increased when the cutoff is increased to 0.5. The upper whisker in the boxplot is raised from 47% to 56%, which indicates slightly more up-regulated genes have been detected.

The peculiarity with the χ^2^ test has to do with the large variability in the results produced by this method, which was already observed in previous simulation studies. The χ^2^ test introduces more false positives than the other methods when the flexible cutoff is increased from 0.3 to 0.5.

As a last rule, we tested combining all methods with the flexible cutoff, which gives results similar to those obtained when the χ^2^ is included in the combination of four methods (Fig. 10).

### Experimentally generated data

Since the simulation studies indicated that the combination of the AC, Fone, Ftwo and Diff methods together with a flexible cutoff was the best approach to detect up-regulated genes, we wanted to test this approach on experimentally generated data.

The data used in these studies originate from cold-stressed *A. thaliana* plants, with a control set of ESTs from non-stressed plants. We also included a set from a subtracted library as a comparison. The sets consist of ESTs originating from pooled cDNA libraries, collected during cold-stress at time points 1 h, 2 h, 5 h, 10 h and 24 h (this also regards the subtracted library), and the sequences were downloaded from dbEST (Boguski et al. 1993) (see Methods for more details on download from dbEST). The main difficulty in this step was to select a proper control set. Since we wanted to exclude the possibility of detecting tissue-specific genes, the control set had to be from the same tissue as used in the stress experiment. We also decided to exclude sets with fewer than 2,000 ESTs, as we regarded those as too small for detecting differentially expressed genes. This ruled out many of the publicly available data sets in dbEST and finally only left the control set that were used in this study.

One of the cold-stress sets was much larger than the other cold-stress set; 22,229 ESTs in comparison to 2,042 ESTs. The set from the subtracted library consisted of 1,250 ESTs and the control set pf 15,790 ESTs. Consequently, this gave the opportunity to investigate the issue of having large differences in sample size.

The main issue in detecting up-regulated genes from EST data concerns the identification of which gene the EST originates from. Commonly, EST analysis follows the sequencing of the tags, with the aim of grouping tags according to their gene origin. This step involves clustering and assembly of the ESTs into contigs and singletons. Optimally, each contig and singleton should represent a unique gene; however, this is not always the case. ESTs have poor sequence quality, some are sequenced from the 5′ end and others from the 3′ end, some genes may be polymorphic, etc. Therefore, the tags from the same gene may not assemble together and tags from different genes may end up in the same contig. This issue has been a topic for decades and there is no real solution to overcome this problem, although several algorithms designed for performing EST analysis with reasonable accuracy have emerged over time.

In this study, the available on-line tool EGassembler (Masoudi-Nejad et al. 2006) was used. This program performs clustering and assembly of the sequences. It also trims low-quality ends and masks sequences that match plastids or mitochondric DNA. The sequences from all sets were concatenated and fed to the program, which was run using default settings.

The next step in identifying which genes are up-regulated is to identify which genes are actually present in the set(s). This is commonly done by performing similarity searches of the contigs and singletons against a database of sequenced genes, such as Blast searches against the nr-database or against a sequenced genome. Like in the EST analysis, this step is also associated with a number of problems. The main difficulty here is to set a proper *E*-value cutoff that will distinguish true matches from false ones.

In this study, we used tBlastx searches with the contigs and singletons against all sequenced genes from *A. thaliana*, and we tested different cutoffs on the *E*-value (data not shown). Setting a more stringent cutoff result in fewer represented genes in the data sets and will also yield a smaller overlap between the different sets. We settled for a cutoff of *E* ≤ 10^−5^, as an attempt to not rule out too many truly expressed genes, but also to keep the number of incorrectly predicted ones at a low level.

The number of contigs and singletons in the two non-subtracted sets were 1,584 and 6,545, respectively, in the subtracted 886, and in the non-stressed set 5,116. However, after the similarity searches (*E* ≤ 10^−5^) the number of genes reduced to 1,133, 5,633, 845 and 3,418, respectively. This means that a number of contigs and singletons matched against the same gene and that some sequences did not receive a significant match. Again, the inherent problems with ESTs apply, which makes it difficult to reliably infer the genes that are actually expressed.

In the simulation studies we recorded if a gene was up-regulated or not, and by that way we could identify which genes were true positives among the detected ones. This provided us with a means to compare the accuracy of the different methods. In order to have the same template when testing on experimental data, we used the collection of cold-induced genes in *A. thaliana* compiled by Hannah et al. (2005) as a gold standard. This collection consists of 4,037 genes reported to be induced in at least two independent microarray experiments and includes both up- and down-regulated genes.

When comparing the genes represented in the sets we could see that an overlap existed, however, not only in the two cold-stressed sets, as expected, but also with the control set. This shows that genes expressed during both normal conditions and during stress responses have been picked up from the cDNA libraries. It can also be deduced that the overlap in the two stress sets is rather low, with only 57% (the smaller set) and 11% (the larger set) of the represented genes in each set. A lower percentage was expected for the larger set, as there are more ESTs, and thereby more genes, in this set. However, one would have expected that almost all genes in the smaller set should be represented in the larger one. These results indicate that a large proportion of different genes have been picked up from the two cDNA libraries. This is most likely due to chance playing a significant role when picking clones, but could also be a result of both technical and biological variations in the cold-stress experiments.

Additionally, some of the genes represented in the control set are also in the collection of cold-regulated genes (Table 3), which means that some of the genes participating in the cold response are also expressed during normal conditions. The total number of cold-regulated genes in the sets are: 312 in the small set (28% of the total in this set), 1,328 in the large set (24%), and in the control set 823 (24%), respectively. This introduces another aspect of the difficulties of detecting differentially expressed genes from EST data. Stress-induced genes may also be expressed and vary in expression during normal conditions, however, at a lower level (Fowler et al. 2005). This will have consequences when detecting cold-regulated genes, since some of these will also have an expression in the control set, which introduces the risk of being ruled out by the detection methods.

**Figure 11:**
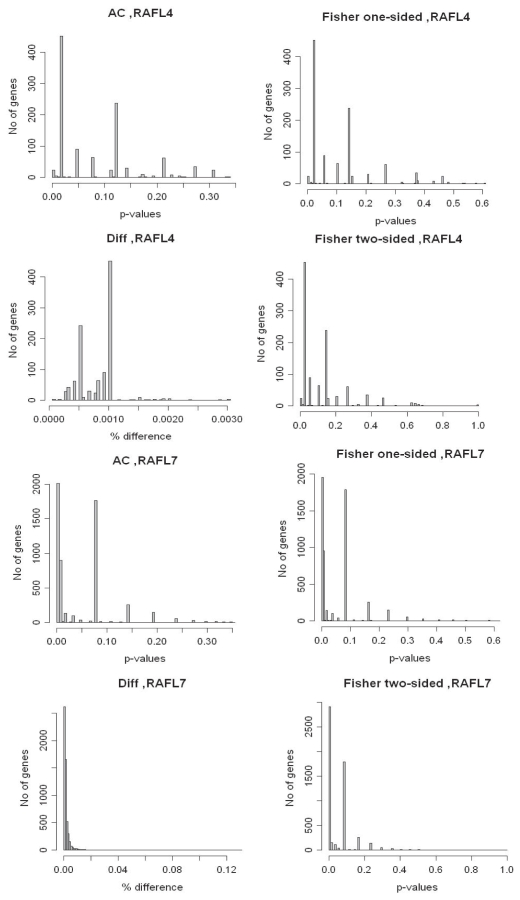
Histograms over the generated statistical test values for the *A. thaliana* cold-induced EST sets RAFL4 and RAFL7, respectively, when compared to a non-stressed set.

In the simulation studies we manually investigated the generated statistical values from the comparisons in order to set proper cutoffs on the test values. For example, we chose *p*-value cutoffs for Fone at 0.1, 0.2, 0.25, 0.4 and 0.5 based on the produced results from the comparisons. In order to set proper cutoff levels for the real data, we took the same approach by manually inspecting the generated results. The histogram in Figure 11 shows the number of genes at different cutoff intervals, when the level is incremented by 0.005 for the AC, Fone and Ftwo and 0.00005 for the difference in percentage regarding the Diff method. This generated a good separation of the genes and, consequently, the cutoff levels were implemented according to this histogram.

**Figure 12:**
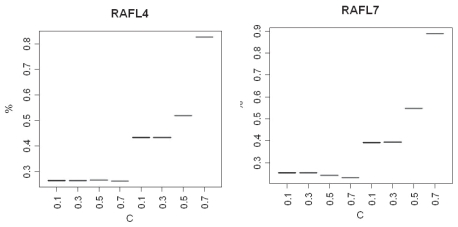
Percentage of true positives and sensitivity when using the combination of methods and a flexible cutoff. The first four levels on the left in each figure show the percentage of true positives, while the remaining levels show the sensitivity.

Finally, we tested different levels for the flexible cutoff and the results can be viewed in Figure 12. For both cold-induced EST sets the level of true positives is very low, ~25% for both sets, irrespectively of the level of the flexible cutoff. This is in disagreement with the simulation studies, where the percentage of true positive was very high at a low flexible cutoff level and, additionally, it decreased with an increased flexible cutoff. On the other hand, the sensitivity increases dramatically when the flexible cutoff increases and reaches to ~85% when *C* = 0.7. However, it should be noticed that the number of false positives also increases, as more genes are detected when the *p*-value becomes more relaxed.

It can also be noticed that the *p*-value cutoffs were relatively stringent for the real data sets. For example, when studying the results of using *C* = 0.7 (worst-case scenario), the *p*-value cutoff level for the AC, Fone and Ftwo methods was *p* ≤ 0.145 regarding the RAFL4 set (smaller) and *p* ≤ 0.085 regarding the RAFL7 set. When setting *C* = 0.1, a *p*-value of ≤0.025 and ≤0.005 for the RAFL4 and RAFL7 set, respectively, was used for both the AC, Fone and Ftwo.

As a comparison, the ESTs picked from the subtracted library corresponded to 845 expressed genes of which 251 (29.7%) had a significant match against a cold-induced gene. This is on a level comparable to the number of stress-induced genes included in the other sets.

## Discussion

Expressed sequence tags (ESTs) offer a relatively quick and cost-effective way of surveying expressed genes during specified conditions, e.g. when searching for genes participating in a stress response or a pathological condition. The identification of differentially expressed genes from this type of data is important when obtaining an understanding of the genetic regulatory machinery underlying the biological processes, as well as for the discovery of molecular markers and potential pharmaceutical targets.

In this study, we compared different methods for detecting differentially expressed genes from pairwise comparisons of EST sets, where one of the sets was generated from the condition under study and the other one served as a control. We generated artificial data and applied different statistical tests; the test statistic proposed by Audic and Claverie, (1997), the Fisher’s one- and two-sided exact test, the χ^2^ test and a method consisting of calculating the difference in relative frequency. We also tested different probability distributions to generate EST data from; the Log normal, Gamma and Poisson distributions. The use of artificial data allowed us to test and evaluate the different methods in a controlled environment. We were especially interested in the situation when the data sets differed in size, were relatively small, and were generated from stress-related conditions.

We conclude from the simulation studies that the results are similar for all probability distributions and that the accuracy of the methods does not rely on a correct assumption regarding the distribution. However, the AC statistic performed slightly better when the Poisson distribution was used, which relates to the fact that this method takes the Poisson distribution into consideration.

Romualdi et al. (2001) investigated the performance of a number of statistical tests when applied to multi-library comparisons with one and two outliers (i.e. genes simulated as differentially expressed in either one or two libraries), and assessed the performance by studying the percentage of false negatives (rather than true positives, as in our study). Although their simulation studies are not entirely comparable to ours, we can still draw some conclusions from them. Their results showed that the general multiple χ^2^ test was the most efficient when applied to multi-library comparisons with two outliers. The χ^2^ 2 × 2 test, which was used in our studies, performed moderately well when applied repeatedly to the multiple libraries, with both one and two outliers. This partly supports the results from our simulation studies, since we investigated the results from simulated pairwise comparisons, which have only one outlier. Consequently, it seems that the general multiple χ^2^ test is better for multi-library comparisons, while the χ^2^ 2 × 2 is not appropriate for either pairwise nor multiple library comparisons.

It is interesting that Fisher’s one-sided and two-sided test performed equally well as the other methods, in contrast to previous studies (Audic and Claverie, 1997; Romualdi et al. 2001). In both Audic and Claverie (1997) and Romualdi et al. (2001) Fisher’s two-sided test was used and the one-sided was not included in the testing. Furthermore, Audic and Claverie (1997) exemplified that Fisher’s two-sided test is always more conservative than AC using a set with only 1,000 ESTs. We agree with this, since the *p*-values for AC is always smaller than for Fone and Ftwo. However, the great difference that was shown by Audic and Claverie (1997) cannot be seen in our results. This might relate to differences in the size of the EST sets, as we used larger sets than Audic and Claverie (1997), but this has to be further analyzed before drawing any conclusions. Additionally, the difference in *p*-values does not tell us anything about the test’s performance, i.e. whether AC is better at separating true positives from false ones because it generates smaller *p*-values. Our results indicate quite the opposite, that the two methods are comparable and that both detect false positives to a similar extent. The deciding factor seems to be the cutoff set for detecting up-regulated genes, and this has been considered in the flexible cutoff.

Romualdi et al. (2001) simulated multi-library comparisons, the results of which indicated that Fisher’s exact test may not be appropriate for such studies. Conversely, we cannot conclude that Fisher’s exact test is inappropriate for detecting differentially expressed genes. We found quite the opposite, that the test is most suitable for this type of analysis. The Fisher’s one-sided test was also used by Ruijter et al. (2002) in a SAGE-simulation experiment, where they reached similar results.

Additionally, it is also interesting that the method of calculating the difference in relative frequency, which is a less rigid statistical method, performs as well as the statistical tests.

As in previous studies, our simulation studies showed that the methods are comparable, but produce slightly different results, which indicate differences in their sensitivity. Since the results from the methods differ, a combination of them might increase the number of detected differentially expressed genes. We therefore investigated this issue more closely by deriving decision trees, and by implementing and testing a range of simple rules. The results from these simulation studies showed that the number of detected up-regulated genes does increase when the AC, Fone and Ftwo and Diff methods are combined. However, the use of the χ^2^ test in combination with any of the other methods introduced more false positives than when it was excluded.

The decision trees also showed that the size of the libraries is of importance when setting a proper cutoff on the test values, since this attribute appeared as a node in the trees. This led to the introduction of a flexible cutoff, which can be used as an alternative to a static one. The advantage of this approach is that the results became less sensitive to the size of the EST sets.

The observations made from the simulation studies were implemented and tested on experimental data, where the ESTs originate from cold stressed *A. thaliana* plants. Other aspects of difficulties in detecting differentially expressed genes from this type of comparison were brought to our attention. These issues relate to the inherent properties of EST data, such as poor sequence quality, polymorphism and unknown gene origin, as well as to the design of the experiments. For example, low abundance of tags from each gene and a lack of proper control sets makes it difficult to relate each EST to the correct original gene, and consequently to derive a correct expression value for each gene. This will have consequences when detecting differentially expressed genes, since the methods rely on correctly derived expression values.

We used a collection of cold-induced genes in *A. thaliana* compiled by Hannah et al. (2005) as gold standard, which provided us with a means to evaluate the accuracy of the combined methods. The methods identified a large number of genes that were not identified in the microarray studies, since the percentage of true positives was very low, irrespective of the threshold set on the flexible cutoff. This may relate to one or several different factors. The cold-induced genes detected by the microarrays may, for various reasons, not be present in the cDNA library. Alternatively, they may not have been picked up from the library. Some of the cold-induced genes were shown to be present in both the cold-stress set and the control set. Since most of the genes have low expression (few ESTs), there might be a lack of significantly differentially expressed genes, which results in many of the genes being discarded by the methods.

There is also the possibility that the two different types of techniques identify different sets of cold-induced genes. Previous studies have shown a limited concordance between tag sampling methods and microarrays (van Ruissen et al. 2005; Haverty et al. 2004; Kavsan et al. 2007). Consequently, this is a possible cause for the low overlap. The EST sequencing technique has a few parameters that affect the results and focuses on expressed genes. However, it is less sensitive than microarrays, since the sets commonly originate from a pooled cDNA library including several time points. Microarray studies give a higher sensitivity, but on the other hand, there are many parameters in the microarray data analysis that can give very different results.

Additionally, Gene Ontology annotation was downloaded from TAIR website, which revealed that many of the false positives are transcription factors, transporters, genes coupled to the photosynthesis, genes with a catalytic activity, transmembrane proteins etc., which are all important for the stress adaptation. A large proportion of the genes are also annotated as either having an unknown molecular function and/or biological process. Hence, the possibility that many of the false positives are cold-induced is apparent, although they have not been annotated as such. There is also the possibility that the collection of cold-induced genes used here as a template may not be the most appropriate.

In conclusion, identifying differentially expressed genes from EST data using relatively small sets is a difficult task. The result is dependent on many steps, which also rely on each other. First, there are the biological experiments that must ensure that the genes participating in the biological process under study are really induced. Thereafter comes the construction of cDNA libraries, which are based on the biological experiments, and picking of clones. This step also introduces another dilemma. The genes may be present in the library, but they may not be picked up, since chance plays such a large role in this step.

Thereafter comes the identification of expressed genes and deriving a correct expression value for each gene. This step is dependent on the quality of the sequences, as well as the abundance of ESTs for each gene, i.e. picking the right clones from the library. The statistical methods rely on correct expression values, and consequently, if the ESTs are not matched to their true genes of origin the methods will detect false positives. The choice of control set is also of importance. For example, if ESTs generated from a different tissue is used as a control, then the genes detected might instead be tissue-specific.

Finally, EST sequencing experiments will generate information on genes participating in a biological process. This is of significant importance when studying processes in organisms that do not have a sequenced genome and where large-scale microarrays are not an option. However, as an alternative of using statistical methods, focused microarrays (also called ‘boutique’-microarrays) constructed on the basis of the EST data could be the next step. The microarrays will increase the sensitivity, compared to EST sequencing, and thereby might give a more reliable result regarding which genes are actually differentially expressed.

## Methods

### Pseudo cDNA libraries

The simulation studies were based on pseudo cDNA libraries and these in turn were based on 20,000 genes following the Log normal, Gamma or Poisson probability distribution, by generating 20,000 random deviates using the *rnorm*, *rgamma* and *rpois* functions, respectively, in the statistical language R.

The generation of pseudo cDNA libraries and the sampling from those were done by using an in-house developed PHP-script. For each of the 20,000 genes, the number generated by the random deviate function was multiplied with 30 and thereafter rounded to the closest integer, so that, e.g. gene *A* was represented by three, gene *B* by one and so on. The results were stored in an array, where each gene was represented as many times as the number generated previously, i.e. gene *A* was represented with three elements in the array, gene *B* with one element, and so on. This resulted in an array containing roughly 1,000,000 elements. From this array a number of samples were randomly picked, using the *mt_rand* function available in PHP.

In the case of the stress-simulated cDNA libraries, 4,000 genes were randomly chosen from the 20,000 genes previously generated, and simulated as up-regulated using two different approaches. In the first approach, the fold-change values followed a normal distribution, with mean *m* = 2 or *m* = 10 and standard deviation *sd* = 1. The random deviate previously generated was multiplied with the absolute value of the randomly drawn sample from the normal distribution. In the second approach, the fold-change value was randomly generated from an exponential probability distribution, with mean *λ* = 1, and the random deviate was multiplied with the value sampled from this distribution.

### ROC and true positive rate curves

For generating Receiver Operating Characteristic (ROC) and true positive rate curves a number of cutoffs were chosen for each method tested and for each cutoff the number of derived true positives (TP), false positives (FP), false negatives (FN) and true negatives (TN) was calculated. ROC curves were thereafter generated by plotting the sensitivity vs. 1-specificity. True positive rate curves were generated by plotting the percentage of TPs vs. the cutoffs.

### Decision trees

The software Weka (Frank et al. 2004) includes a range of machine learning algorithms for data mining problems, such as classification, clustering and association rules. The Weka implementation of the tree induction algorithm J48 was applied to simulated data and used for deriving decision trees, with default settings.

For these simulation studies, the up-regulated genes were simulated with a fold-change sampled from the exponential distribution, with mean *λ* = 1. A random number of transcripts were sampled from each library, and for each pairwise comparison all methods were applied without any cutoff. The sampling and pairwise comparison was conducted 20 times, each time randomly choosing genes as up-regulated, as well as setting their fold-change to a random one selected from the exponential distribution.

The results from the simulations, together with the recorded class, i.e. whether a gene was simulated as up-regulated or normally expressed, were concatenated and prepared in a format which the Weka software could read. It was thereafter fed to the J48 program, using default values on the parameters and without any cross-validation. The whole procedure was repeated five times for each distribution, thus generating five decision trees for each distribution.

### Test rules

A range of simple rules were implemented in a PHP-script and tested on the simulated data. In these simulation studies, we also tested different combinations of sample sizes. The sample sizes were divided into three categories: small (2,000–4,000 ESTs); medium (5,000–7,000 ESTs); and large (8,000–10,000 ESTs). A random number of transcripts were sampled within each range, using the *mt_rand* function in PHP.

All combinations of sample sizes from each library were tested, and for each combination the sampling was repeated three times. In addition, for each pairwise comparison different genes were simulated as differentially expressed up-regulated, as well as their fold-changes. The rules were applied to each pairwise comparison and the results from each rule tested were plotted in a boxplot using the statistical language R.

### EST analysis and gene identification

EST sequences were downloaded from dbEST, using the following search phrases: ‘RAFL4’, ‘RAFL7’, ‘RAFL18’ and ‘Arabidopsis AND aboveground organs’, which relate to the two cold-stress sets, the subtracted cold-stress set and the control set used, with UniGene Lib. IDs 10438, 10441, 10433, and 5335, respectively. The sequences from all sets were concatenated and the on-line tool EGassembler (Masoudi-Nejad et al. 2006) was used for EST analysis, with default settings. Identification of expressed genes was done by a tBlastx search, using an *E* ≤ 10^−10^, against the TAIR6 Genome Release of *Arabidopsis* gene sequences.

## Supplementary Materials

Figure S1 ROC curves for an up-regulation of either 2-fold (figures on the left) or 10-fold changes (figures on the right). Solid and dashed lines indicate 2,000 and 10,000 sampled ESTs, respectively. In a–b) the transcript abundance followed a Gamma distribution and in c–d) a Poisson distribution was used. Black: difference in relative frequency, red: χ^2^, green: AC and violet: Fone, blue: Ftwo.

Figure S2 Shows the percentage true positives detected by the different statistical methods versus the number of detected genes. Here, the transcript abundance followed a Gamma distribution and the size of the EST sets differed in the treatment and control set. Black: Diff, green: AC, violet: Fone, blue: Ftwo, red: χ^2^. The circles correspond to different cutoffs (see Table).

Figure S3 Shows the percentage true positives detected by the different statistical methods versus the number of detected genes. Here, the transcript abundance followed a Gamma distribution and the size of the EST sets differed in the treatment and control set. Black: Diff, green: AC, violet: Fone, blue: Ftwo, red: χ^2^. The circles correspond to different cutoffs (see Table).

Figure S4 Excerpt from a file of input data to the induction algorithm J48 available in the Weka program (version).

Figure S5 Example of decision trees generated by the induction algorithm J48 available in the Weka program (version).

## Figures and Tables

**Table 1:** Cutoffs used when detecting up-regulated genes and are marked as circles in the true positive rate curve figures.

Diff	χ	AC	Fone	Ftwo
0.002	0.01	0.01	0.01	0.01
0.001	0.05	0.05	0.05	0.05
0.0005	0.1	0.1	0.1	0.1
0.0003	0.2	0.15	0.2	0.2
0.0002	0.4	0.2	0.4	0.4
0.0001	0.5		0.5	0.5

**Table 2:** These rules implemented and tested on the simulated data. *C* relates to the flexible cutoff used in the detection of up-regulated genes. See Figure 7 for pseudocode on how *C* is derived.

No	Rule
1	IF AC ≤ 0.16
2	IF AC ≤ 0.18
3	IF AC ≤ 0.19
4	IF AC ≤ 0.20
5	IF Fone ≤ 0.1
6	IF Fone ≤ 0.2
7	IF Fone ≤ 0.25
8	IF Fone ≤ 0.4
9	IF Fone ≤ 0.5
10	IF Ftwo ≤ 0.1
11	IF Ftwo ≤ 0.2
12	IF Ftwo ≤ 0.25
13	IF Ftwo ≤ 0.4
14	IF Ftwo ≤ 0.5
15	IF χ^2^ ≤ 0.1
16	IF χ^2^ ≤ 0.2
17	IF χ^2^ ≤ 0.4
18	IF χ^2^ ≤ 0.5
19	IF Diff ≥ 0.0003
20	IF Diff ≥ 0.00025
21	IF Diff ≥ 0.0002
22	IF Diff ≥ 0.0001
23	IF AC ≤ *C*
24	IF Fone ≤ *C*
25	IF Ftwo ≤ *C*
26	IF Diff ≥ *C*
27	IF χ^2^ ≤ *C*
28	IF AC ≤ *C* OR Fone ≤ *C* OR Ftwo ≤ *C* OR Diff ≥ *C*
29	IF AC ≤ *C* OR Fone ≤ *C* OR Ftwo ≤ *C* D OR χ^2^ ≤ *C*
30	IF AC ≤ *C* OR Fone ≤ *C* OR Diff ≥ *C* OR χ^2^ ≤ *C*
31	IF AC ≤ *C* OR χ^2^ ≤ *C* OR Ftwo ≤ *C* OR Diff ≥ *C*
32	IF χ^2^ ≤ *C* OR Fone ≤ *C* OR Ftwo ≤ *C* OR Diff ≥ *C*
	IF AC ≤ *C* OR χ^2^ ≤ *C* OR Fone ≤ *C* OR Ftwo ≤ *C* OR Diff ≥ *C*

See text and Methods for how *C* is computed.

**Table 3:** Comparison of the number of cold-induced genes in the different EST sets, according to a tBlastx search against *A. thaliana* genes using an *E*-value cutoff of 10^−5^. EST set: ‘RAFL4’ and ‘RAFL7’ are the cold stressed non-subtracted libraries and ‘Control’ is the non-stressed library; Total: the number of genes with a significant match to at least one EST; Cold-induced: the number of genes in the sets reported by Hannah et al. (2005) as cold-inducible.

EST set	Total	Cold-induced
RAFL4	1133	312 (28%)
RAFL7	5633	1328 (24%)
Control	3418	823 (24%)
