# Supplementary figures and images for: Evaluation of Combining Several Statistical Methods with a Flexible Cutoff for Identifying Differentially Expressed Genes in Pairwise Comparison of EST Sets
Source: Bioinform Biol Insights. 2008 May 1;2:215–37. doi: 10.4137/bbi.s431 (PMC2735943; doi:10.4137/bbi.s431)

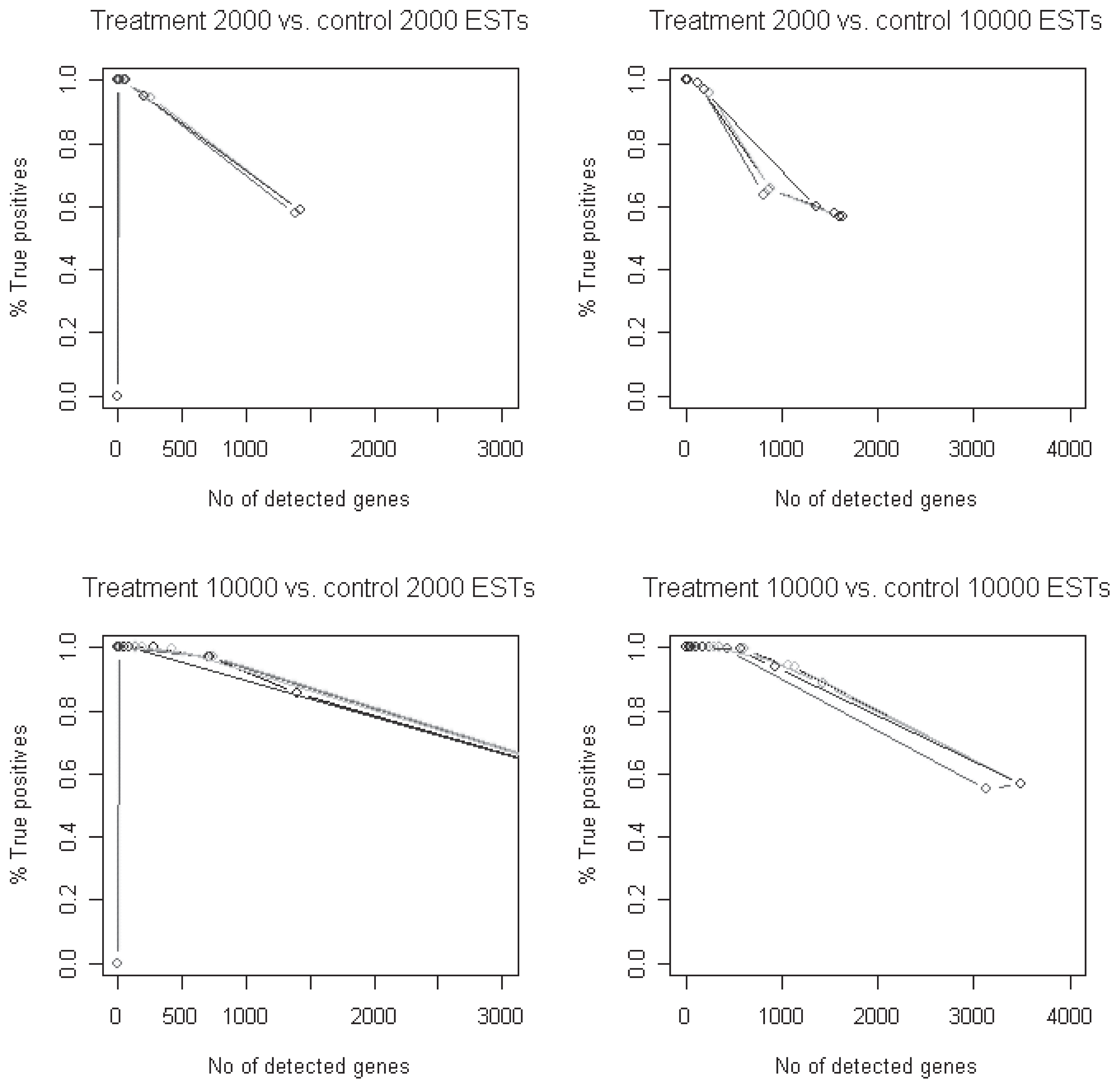

Supplement: Figure S1 [file bbi-2008-215f14.tif]

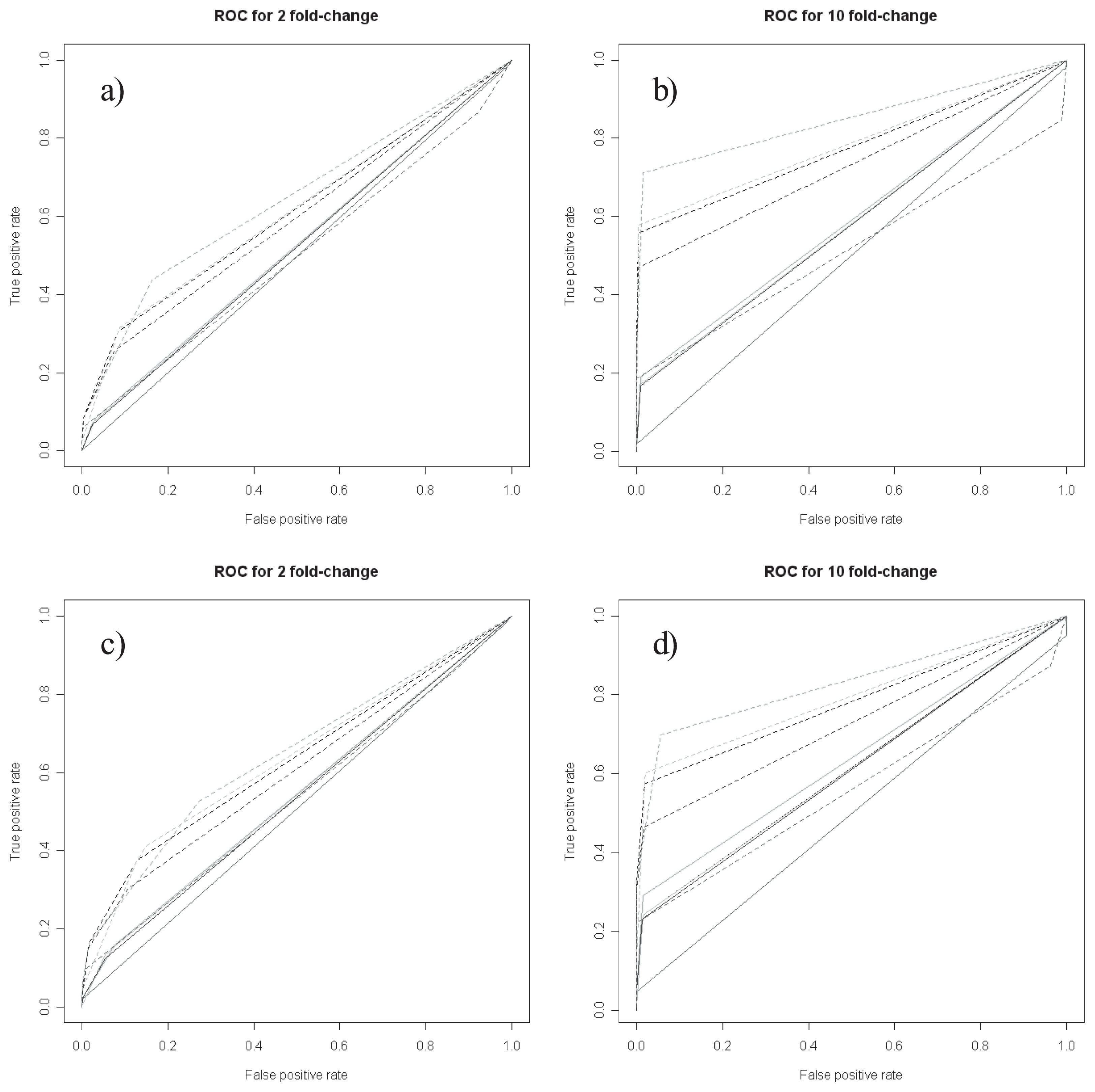

Supplement: Figure S2 [file bbi-2008-215f13.tif]

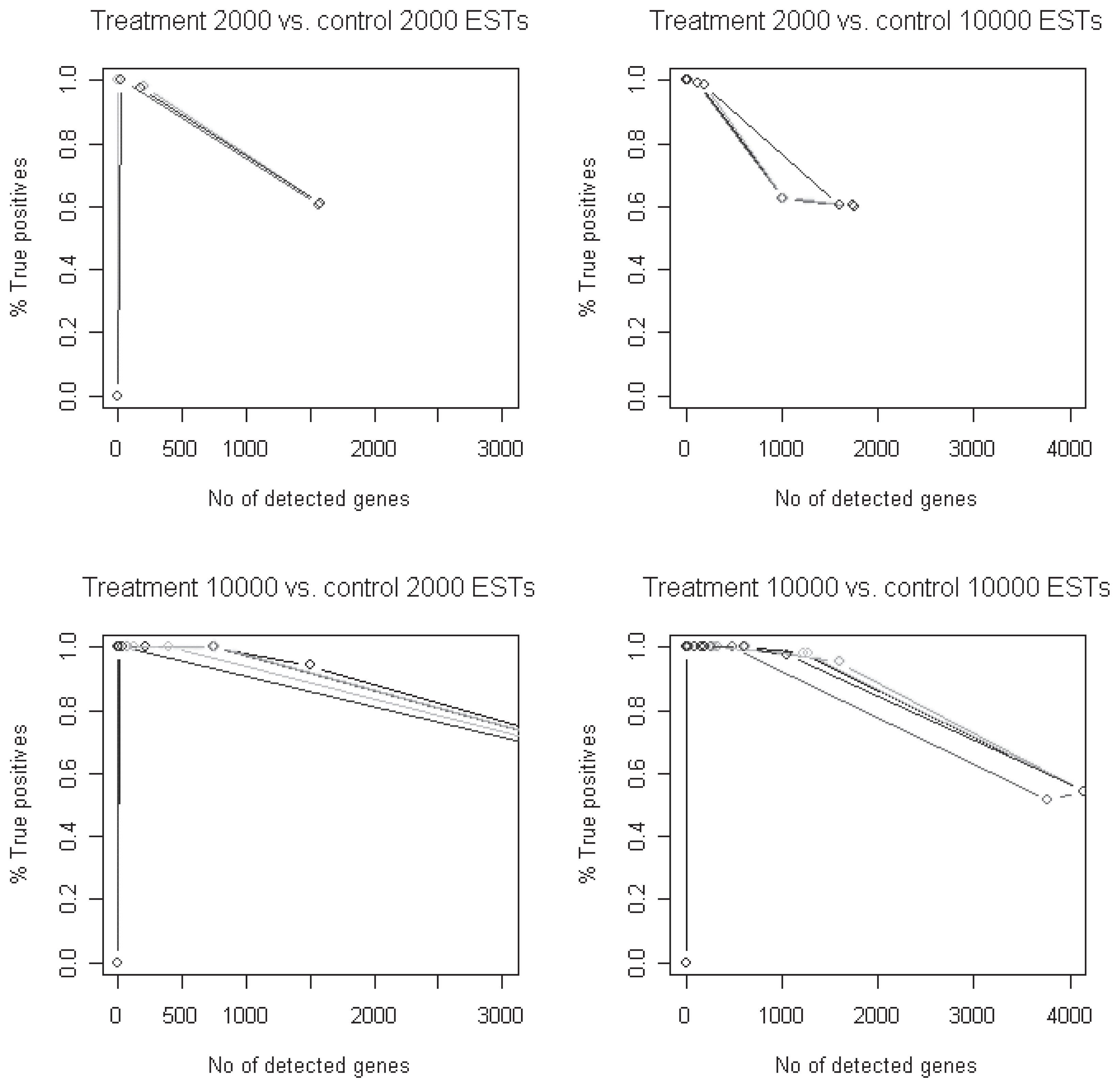

Supplement: Figure S3 [file bbi-2008-215f15.tif]

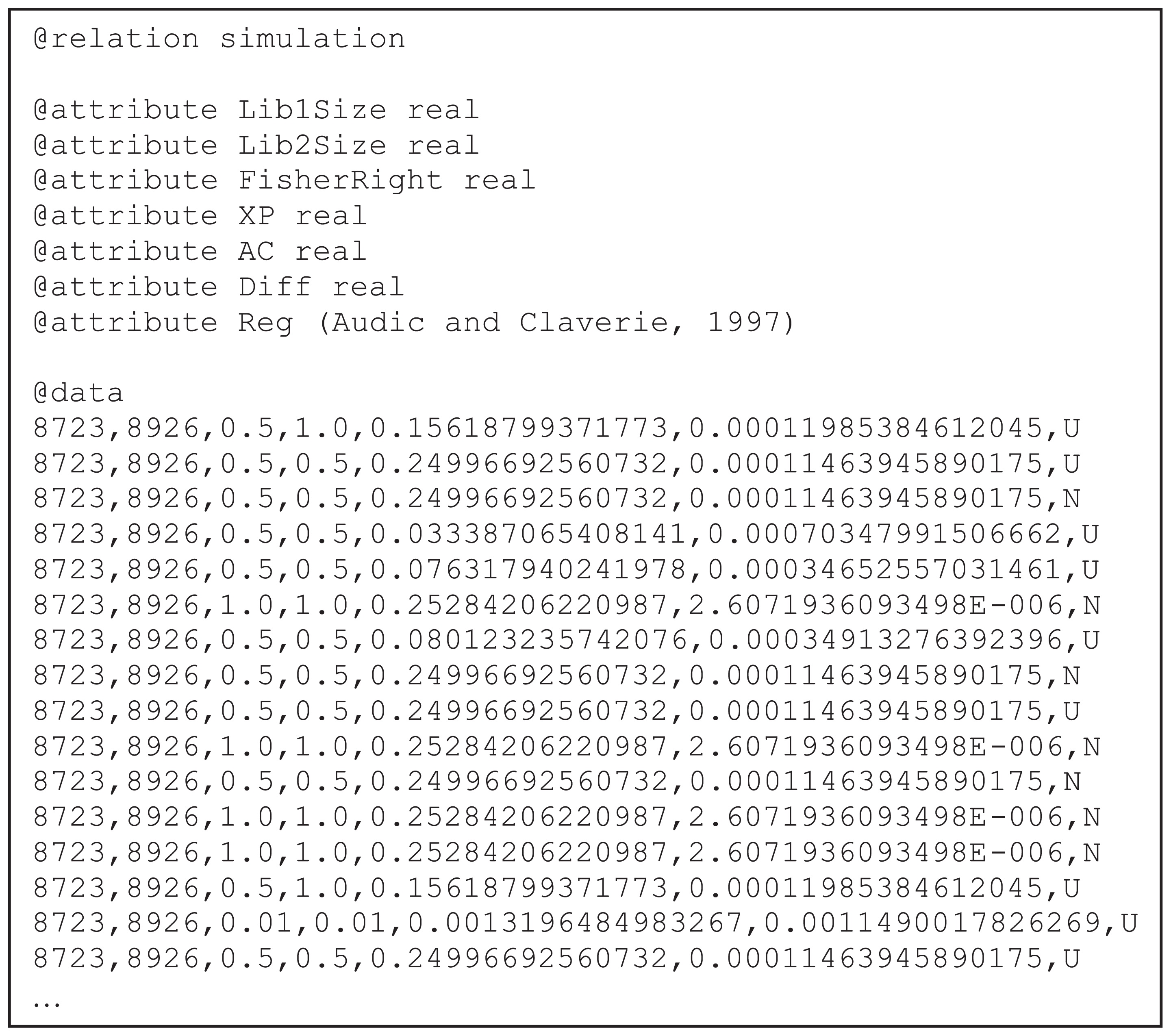

Supplement: Figure S4 [file bbi-2008-215f16.tif]

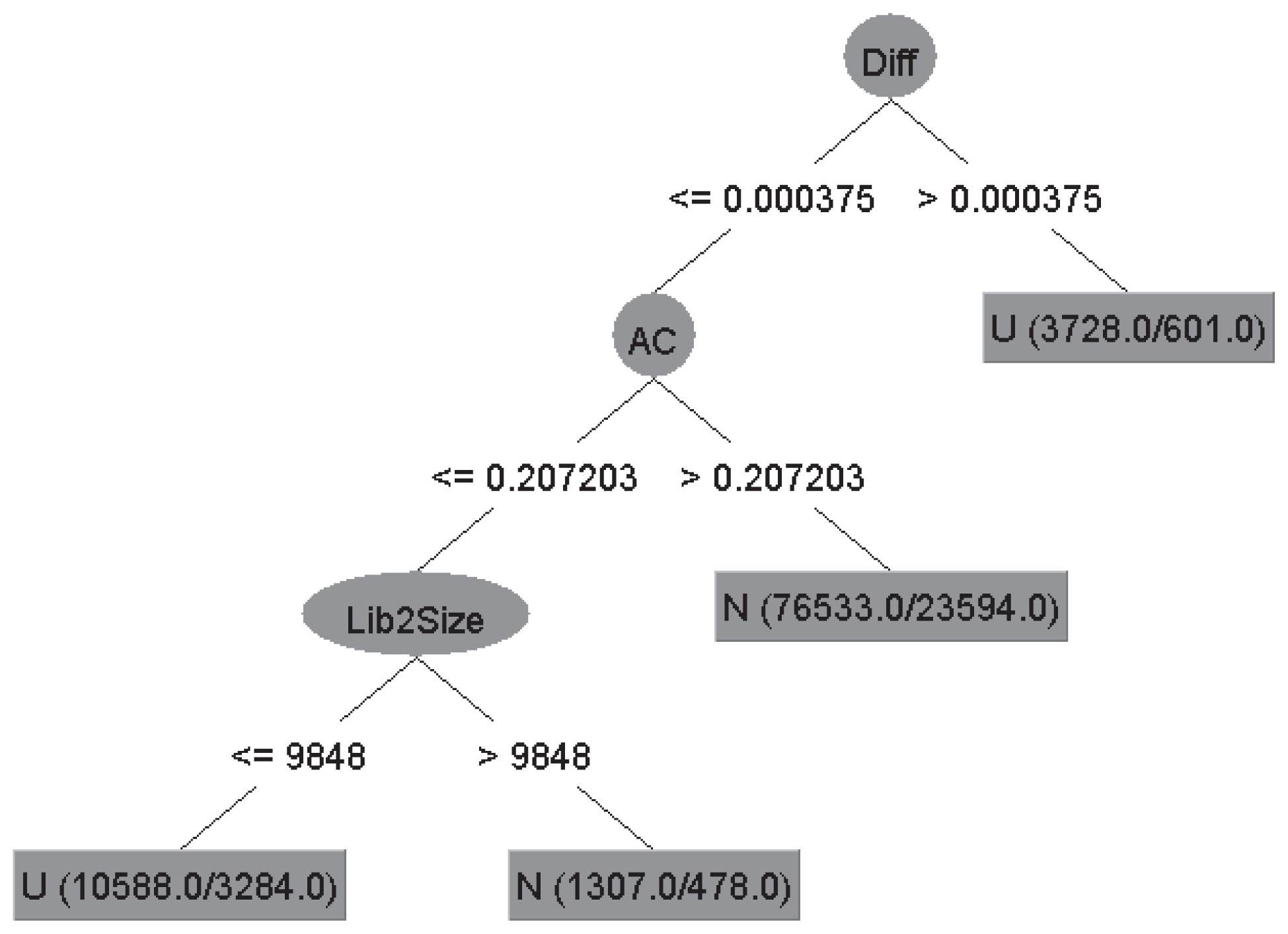

Supplement: Figure S5 [file bbi-2008-215f17.tif]
